# Endoscopic resection with one-port placement for duodenal neuroendocrine tumor: toward more minimally invasive endoscopic management

**DOI:** 10.1055/a-2516-2823

**Published:** 2025-02-05

**Authors:** Elsayed Ghoneem, Osamu Dohi, Tomoko Ochiai, Naoto Iwai, Naohisa Yoshida, Kazuya Takabatake, Takeshi Kubota

**Affiliations:** 1 Gastroenterology and Hepatology Department, Mansoura University, Mansoura, Egypt; 2 Molecular Gastroenterology and Hepatology, Graduate School of Medical Science, Kyoto Prefectural University of Medicine, Kyoto, Japan; 3 Division of Digestive Surgery, Department of Surgery, Kyoto Prefectural University of Medicine, Kyoto, Japan

Endoscopic resection with one-port placement (EROPP) was initially reported as a hybrid endoscopic full-thickness resection procedure, offering a safe and minimally invasive treatment option for gastric gastrointestinal stromal tumors 1 . A key advantage of EROPP is its ability to maintain constant intra-abdominal pressure and ensure a clear visual field during endoscopy 1 . This report presents the first use of EROPP in treating a small duodenal neuroendocrine tumor (NET) in a patient for whom major surgery was high risk.

A 75-year-old man with a medical history of diabetes mellitus and surgical treatment for colorectal cancer and lung cancer was referred for management of a 10-mm NET at the duodenal bulb ( Fig. 1 ). Given the patient’s advanced age, comorbidities, and previous extensive surgical history, EROPP was selected as the treatment modality. The procedure was performed using a single laparoscopic port inserted into the left abdominal region before the endoscopic procedure ( Fig. 2 **a** , Video 1 ).

**Fig. 1:**
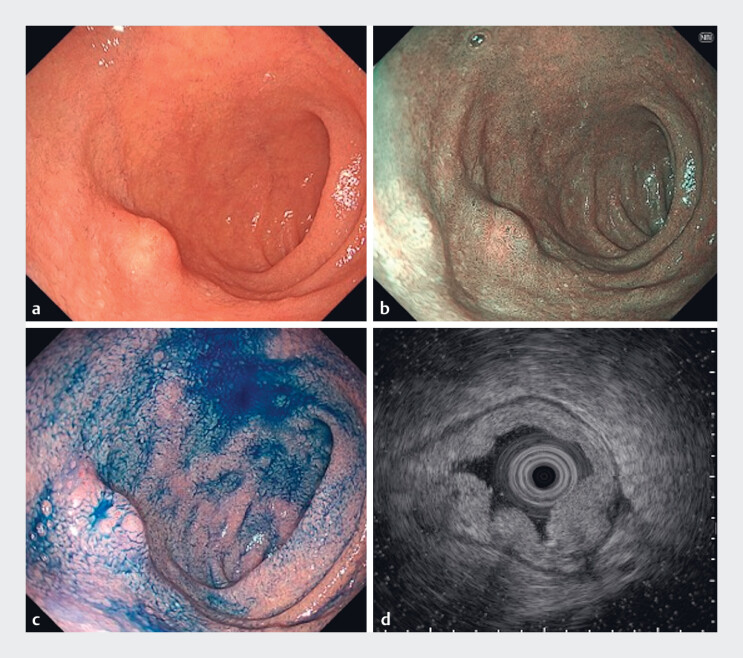
A 10-mm neuroendocrine tumor with submucosal invasion at the duodenal bulb. **a** White-light imaging. **b** Narrow-band imaging. **c** Chromoendoscopy with indigo carmine. **d** Endoscopic ultrasonography.

**Fig. 2:**
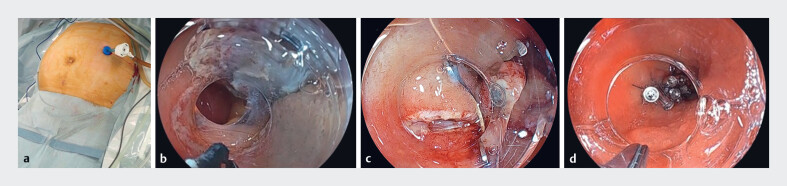
Endoscopic resection with one-port placement. **a** A single laparoscopic port inserted into the left abdominal region. **b** Complete full-thickness resection using a Clutch Cutter (Fujifilm Co., Tokyo, Japan). **c** Incomplete closure of the defect after using an over-the-scope clip. **d** Complete closure of the defect using an over-the-scope clip with reopenable clip-over-the-line method.

Endoscopic full-thickness resection with one-port placement for a duodenal neuroendocrine tumor.Video 1

The endoscopic procedure was performed, starting with marking around the lesion and submucosal injection into the submucosal layer, followed by circumferential incision using a 3.5-mm Clutch Cutter (Fujifilm Co., Tokyo, Japan) under gel immersion. The submucosal fibers were trimmed exposing the muscle layer, and dental floss and clip traction were applied. Then, the gel was removed to prevent exposing the peritoneum to the gel. Complete full-thickness resection and specimen retrieval perorally were accomplished in 25 minutes ( Fig. 2 **b** ). Closure of the duodenal defect was then attempted using a 9-mm over-the-scope (OTS) clip. Unfortunately, the OTS clip failed to close the defect completely, and fluid leakage was detected from the laparoscopic port ( Fig. 2 **c** ). Complete defect closure was achieved using the reopenable clip-over-the-line method ( Fig. 2 **d** ) 2 .

The patient was discharged on the 4th day after the procedure with an uneventful recovery. Histopathological diagnosis was a NET G1 with negative horizontal and vertical margins but positive for lymphovascular invasion.

Endoscopy_UCTN_Code_TTT_1AO_2AG_3AF

## Notes

Endoscopy E-Videos https://eref.thieme.de/e-videos
*E-Videos* is an open access online section of the journal *Endoscopy* , reporting on interesting cases and new techniques in gastroenterological endoscopy. All papers include a high-quality video and are published with a Creative Commons CC-BY license. Endoscopy E-Videos qualify for HINARI discounts and waivers and eligibility is automatically checked during the submission process. We grant 100% waivers to articles whose corresponding authors are based in Group A countries and 50% waivers to those who are based in Group B countries as classified by Research4Life (see: https://www.research4life.org/access/eligibility/ ). This section has its own submission website at https://mc.manuscriptcentral.com/e-videos .

## References

[1] Sawada A Hirasawa K Sato C Endoscopic resection with one-port placement: a newly developed technique for the safe management of advanced endoscopic resection for gastric gastrointestinal stromal tumors Digestion 2023 104 460 467 37647880 10.1159/000532012 PMC10711755

[2] Nomura T Sugimoto S Temma T Reopenable clip-over-the-line method for closing large mucosal defects following colorectal endoscopic submucosal dissection: a feasibility study Endosc Int Open 2023 11 E697 E702 10.1055/a-2150-5840 37564328 PMC10411209

